# Supplementary material for: The dimer-dependent catalytic activity of RAF family kinases is revealed through characterizing their oncogenic mutants
Source: Oncogene. 2018 Jun 21;37(43):5719–34. doi: 10.1038/s41388-018-0365-2 (PMC6202329; doi:10.1038/s41388-018-0365-2)
Supplement: Supplementary file 1 — Supplementary information [file 41388_2018_365_MOESM1_ESM.pdf]

Supplementary Information:

Table S1. The Conserved Motifs/Residues of RAF Family Kinases Regulating Their Activity

Raf Isoform	NtA motif	Catalytic spine mutation	Regulatory spine mutation	Central RH alteration in dimer interface	APE motif
ARAF	$_{299}$SGYY$_{302}$	V324F	L358M	R362H	$_{474}$AAE$_{476}$
BRAF	$_{446}$SSDD$_{449}$	V471F	L505M	R509H	$_{621}$APE$_{623}$
CRAF	$_{338}$SSYY$_{341}$	V363F	L397M	R401H	$_{513}$APE$_{515}$

Table S1. The Conserved Motifs/Residues of RAF Family Kinases Regulating Their Activity. The key motifs/residues of RAF family kinases involved in this study were listed in this table.

Table S2. Protein Kinase Mutants With In-frame Deletions of β3-αC Loop in Cancers

Kinase	Sequence (β3 … αC)	No.
**ARAF**	**VAVKVLKVSQPTAEQAQAFKNEMQVLRKTR**	
	VAVKVLKVSQPTAE--QAFKNEMQVLRKTR	1
**BRAF**	**VAVKMLNVTAPTPQQLQAFKNEVGVLRKTR**	
	VAVK---VTAPTPQQLQAFKNEVGVLRKTR	1
	VAVKM------TPQQLQAFKNEVGVLRKTR	1
	VAVKMY-----TPQQLQAFKNEVGVLRKTR	1
	VAVKMF-----TPQQLQAFKNEVGVLRKTR	1
	VAVKML-----TPQQLQAFKNEVGVLRKTR	6
	VAVKML------PQQLQAFKNEVGVLRKTR	1
	VAVKMLK-----PQQLQAFKNEVGVLRKTR	1
	VAVKMLN-----PQQLQAFKNEVGVLRKTR	2
	VAVKMLNA-----QQLQAFKNEVGVLRKTR	1
	VAVKMLNV-----QQLQAFKNEVGVLRKTR	2
	VAVKMLNVK-----QLQAFKNEVGVLRKTR	2
	VAVKMLNVTA-----LQAFKNEVGVLRKTR	2
**JAK1 DomainB**	**VILKVLDPS---HRDISLAFFEAASMMRQV**	
	VILKVW--------DISLAFFEAASMMRQV	2
	VILKVLDPS---H--ISLAFFEAASMMRQV	2
	VILKVLDPS---HRDISLA-FEAASMMRQV	1
**ERBB2**	**VAIKVLRENT-SPKANKEILDEAYVMAGVG**	
	VAIKV------SPKANKEILDEAYVMAGVG	1
	VAIKVA------PKANKEILDEAYVMAGVG	1
**EGFR**	**VAIKELREAT-SPKANKEILDEAYVMASVD**	
	VALKR---AT-SPKANKEILDEAYVMASVD	1
	VAVK-----T-SPKANKEILDEAYVMASVD	1
	VAI---REAT-SPKANKEILDEAYVMASVD	1
	VAI-----AT-SPKANKEILDEAYVMASVD	14
	VAI------T-SPKANKEILDEAYVMASVD	19
	VAIT-----T-SPKANKEILDEAYVMASVD	4
	VAIK-LREAT-SPKANKEILDEAYVMASVD	2
	VAIK---EAT-SPKANKEILDEAYVMASVD	2
	VAIK----AT-SPKANKEILDEAYVMASVD	13
	VAIKY---AT-SPKANKEILDEAYVMASVD	1
	VAIK-----T-SPKANKEILDEAYVMASVD	3489
	VAIKX----T-SPKANKEILDEAYVMASVD	6
	VAIKXX---T-SPKANKEILDEAYVMASVD	24
	VAIK------SPKANKEILDEAYVMASVD	29
	VAIKX------SPKANKEILDEAYVMASVD	79
	VAIKXX-----SPKANKEILDEAYVMASVD	24
	VAIK--------PKANKEILDEAYVMASVD	16
	VAIKX-------PKANKEILDEAYVMASVD	152
	VAIKIV------PKANKEILDEAYVMASVD	2
	VAIK---------KANKEILDEAYVMASVD	5
	VAIKXX-------KANKEILDEAYVMASVD	13
	VAIK-----------NKEILDEAYVMASVD	1
	VAIKE-REAT-SPKANKEILDEAYVMASVD	3
	VAIKE--EAT-SPKANKEILDEAYVMASVD	1
	VAIKE---AT-SPKANKEILDEAYVMASVD	28
	VAIKEX--AT-SPKANKEILDEAYVMASVD	2
	VAIKE----T-SPKANKEILDEAYVMASVD	14
	VAIKEX---T-SPKANKEILDEAYVMASVD	184
	VAIKE------SPKANKEILDEAYVMASVD	199
	VAIKEX-----SPKANKEILDEAYVMASVD	73
**EGFR**	**VAIKELREAT-SPKANKEILDEAYVMASVD**	
	VAIKEPT----SPKANKEILDEAYVMASVD	1
	VAIKE-------PKANKEILDEAYVMASVD	97
	VAIKEX------PKANKEILDEAYVMASVD	7
	VAIKEXX-----PKANKEILDEAYVMASVD	4
	VAIKE--------KANKEILDEAYVMASVD	33
	VAIKEX-------KANKEILDEAYVMASVD	321
	VAIKECG------KANKEILDEAYVMASVD	1
	VAIKE---------ANKEILDEAYVMASVD	3
	VAIKEN--------ANKEILDEAYVMASVD	1
	VAIKEXX-------ANKEILDEAYVMASVD	2
	VAIKEANKG-----ANKEILDEAYVMASVD	1
	VAIKE----------NKEILDEAYVMASVD	1
	VAIKEXX--------NKEILDEAYVMASVD	3
	VAIKESKG-------NKEILDEAYVMASVD	5
	VAIKESKDD-------KEILDEAYVMASVD	1
	VAIKEL------PKANKEILDEAYVMASVD	1
	VAIKEL-------KANKEILDEAYVMASVD	2
	VAIKELRQ-----KANKEILDEAYVMASVD	1
	VAIKELRQP--------EILDEAYVMASVD	2
	VAIKELRE------ANKEILDEAYVMASVD	2
	VAIKELRE---------EILDEAYVMASVD	2
	VAIKELRE----------ILDEAYVMASVD	2
	VAIKELREP---------ILDEAYVMASVD	3
	VAIKELREXX---------LDEAYVMASVD	2
	VAIKELREA---------ILDEAYVMASVD	5
	VAIKELREAA--------ILDEAYVMASVD	1
	VAIKELREA----------LDEAYVMASVD	2
	VAIKELREAX---------LDEAYVMASVD	22
	VAIKELREAA-C-------LDEAYVMASVD	1
	VAIKELREAX-XX------LDEAYVMASVD	3
	VAIKELREAT---------LDEAYVMASVD	27
	VAIKELREAT------------------D	1
	VAIKELREAT-S-------LDEAYVMASVD	1
	VAIKELREAT-SP------LDEAYVMASVD	1
	VAIKELREAT-SPKA-----DEAYVMASVD	1

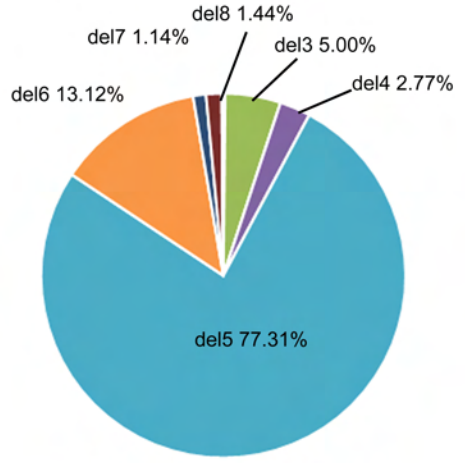

Frenquency of EGFR mutants

Table S2. Protein Kinase Mutants With In-frame Deletions of β3-αC Loop in Cancer. To explore all protein kinase mutants with variable β3-αC loop deletions in cancer genomes, we we interrogated the ICGC (International Cancer Genome Consortium) database, the cBioportal for Cancer Genomics database, and the COSMIC (Catalogue of Somatic Mutations in Cancer) database. All known mutations including those reported in literatures[1-4] were summarized in this table.

Figure S1

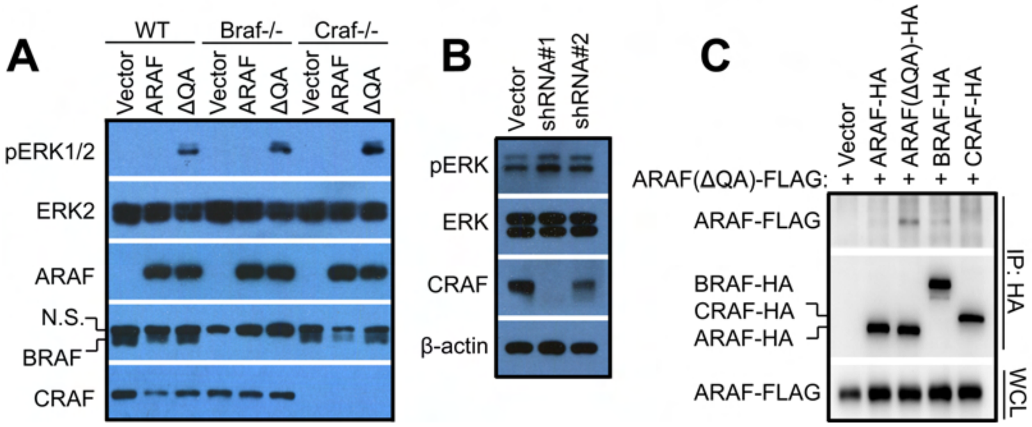

Figure S1 (related to Figure 1). ARAF mutant with Q347A348 deletion is activated by elevated homodimerization but not heterodimeization.
A, ARAF(ΔQA) and its wild-type counterpart were stably expressed in RAF-knockout fibroblasts, and their activity was measured by anti-phosphoERK1/2 immunoblot. ARAF(ΔQA) activates MEK-ERK signaling in wild-type, BRAF-/-, and CRAF-/- MEFs. B, the CRAF knockdown does not affect the activity of ARAF(ΔQA) in BRAF-/- MEFs. The activity of ARAF(ΔQA) in BRAF-/- with or without shRNAs against CRAF was measured as phosphoERK by immunoblot. C, ARAF(ΔQA) hardly heterodimerizes with wild-type ARAF, BRAF and CRAF. The wild-type RAF molecules were coexpressed with ARAF(ΔQA) in 293T cells, and the coimmunoprecipitation assay was carried out as described before[5]. All images are representative of at least three independent experiments.

Figure S2

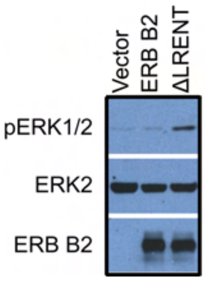

Figure S2. An ERB B2 mutant with β3-αC loop deletion has constitutive activity.
The ERB B2 mutant with in-frame β3-αC loop deletion was expressed in 293T cells and its activity was measured by anti-phosphoERK1/2 immunoblot. All images are representative of at least three independent experiments.

Figure S3

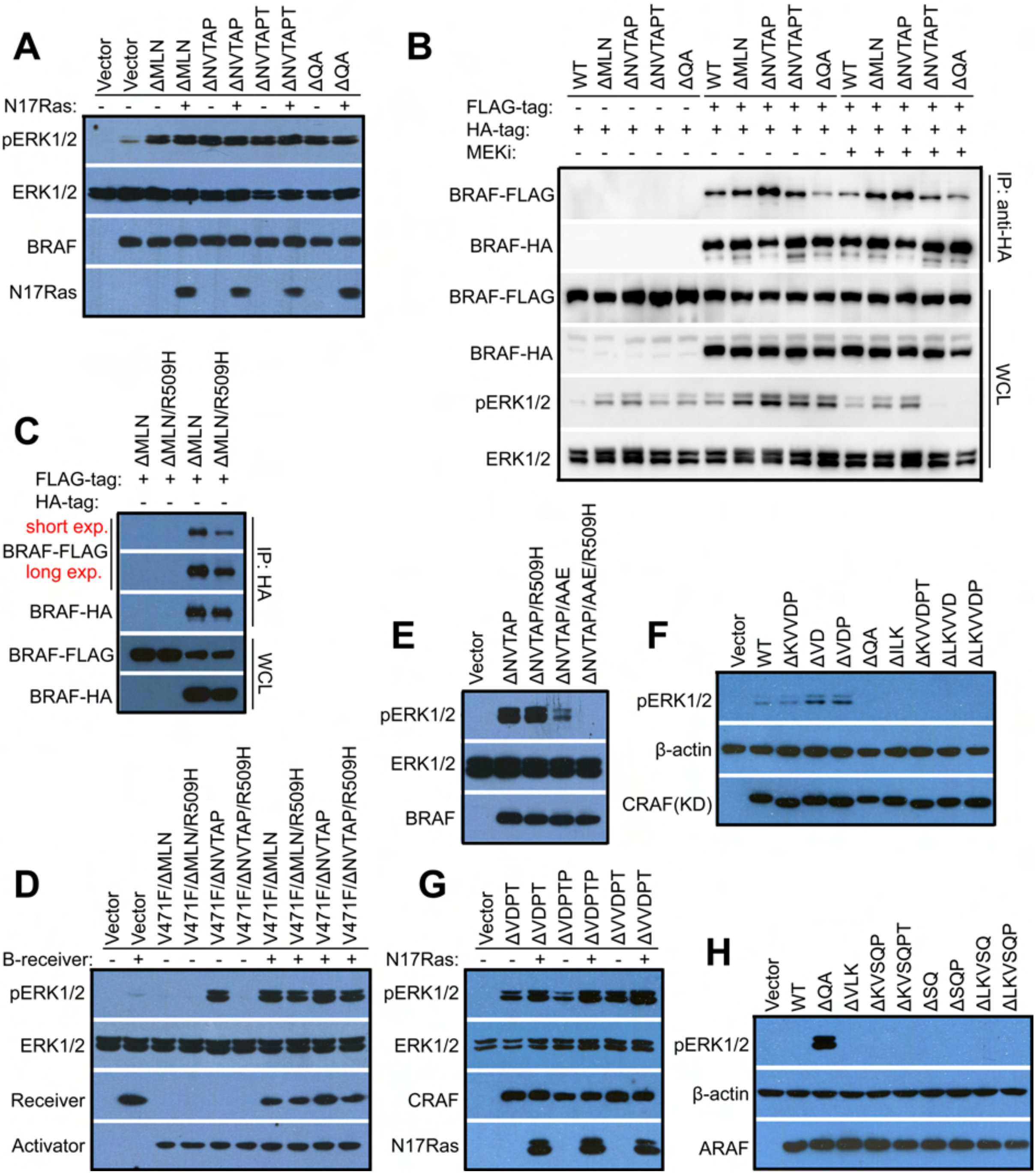

Figure S3 (related to Figure 4). RAF kinase is activated by in-frame β3-αC loop deletions through enhanced homodimerization.
(A) BRAF mutants with in-frame β3-αC loop deletions exhibit a RAS-independent activity. The BRAF mutants were expressed with or without N17RAS in 293T cells and their activity was measured by anti-phosphoERK1/2 immunoblot.
(B-C) BRAF mutants with in-frame β3-αC loop deletions have increased albeit variable dimer affinity. The dimer affinity of BRAF mutants in 293T transfectants treated with or without MEK inhibitor was measured by co-immunoprecipitation as in Figure 1E.
(D) The central R509H alteration in dimer interface is not able to abolish the allosteric activity of Catalytic spine-fused BRAF mutants with a high dimer affinity toward BRAF-receiver. The RAF co-activation assay was carried out as described before[5,6].

(E) The combined alteration of APE motif and the central Arg in dimer interface abolishes the activity of BRAF mutant with a high dimer affinity. The BRAF mutants were expressed in 293T cells and their activity was measured by anti- phosphoERK1/2 immunoblot.

(F-G) In-frame β3-αC loop deletions turn on CRAF independent of RAS. The activity of CRAF mutants expressed in 293T cell with or without N17RAS was measured as in (A).

(H) ARAF is activated by ΔQA but not other deletions in β3-αC loop. The activity of ARAF mutants expressed in 293T cells was measured as in (A).

All images are representative of at least three independent experiments.

Figure S4

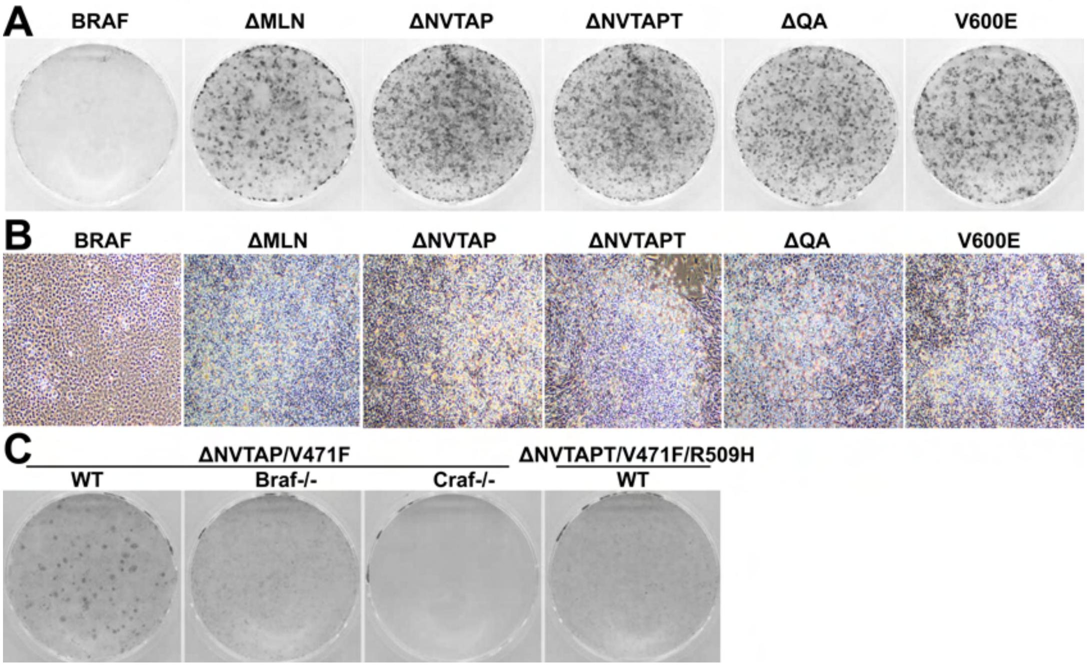

Figure S4 (related to Figure 5). BRAF mutants with in-frame β3-αC loop deletions have a strong oncogenic potential.

(A-B) BRAF mutants with in-frame β3-αC loop deletions transform both BRAF-knockout and CRAF-knockout immortalized fibroblasts. A, foci formation assay of immortalized $braf^{-/-}$ MEFs that express BRAF mutants. B, cellular morphology images of immortalized $craf^{-/-}$ MEFs that express BRAF mutants.

(C) The transforming ability of kinase-dead BRAF(ΔNVTAP/V471F) depends on endogenous RAF molecules and dimerization. Foci formation assay of immortalized MEFs that express BRAF mutant was carried out as in Figure 1C.

All images are representative of at least three independent experiments.

Figure S5

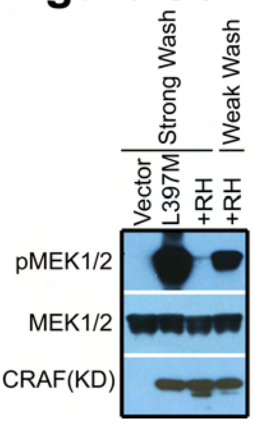

Figure S5 (related to Figure 6). The constitutively active CRAF R-spine mutant with a low dimer affinity retains partial catalytic activity in vitro upon purification with a gentle wash of PBS. CRAF mutants were expressed in 293T cells and purified by immunoprecipitation with either a strong wash of RIPA buffer (1% NP-40) or a gentle wash of PBS. Then their activity was measured by in vitro kinase assay as in Figure 6.
All images are representative of at least three independent experiments.

Figure S6

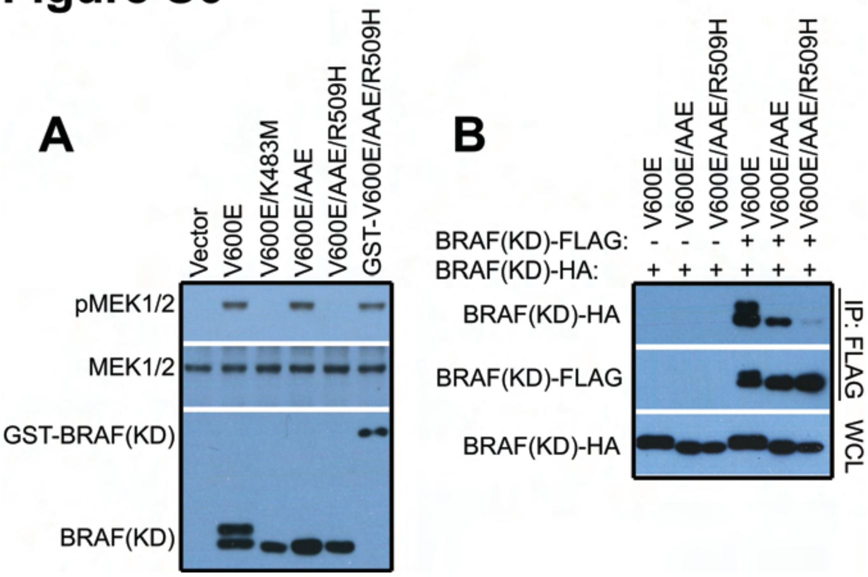

Figure S6 (related to Figure 7). The BRAF(V600E) mutant with non-canonical APE motif has constitutive activity but is sensitive to RH alteration in dimer interface.
(A) The BRAF(V600E) mutant with non-canonical APE motif can phosphorylate MEK as well as its prototype, which is blocked by further RH alteration in dimer interface. BRAF mutants were expressed in 293T cells and the phospho-MEK1/2 was detected by immunoblot. The kinase-dead BRAF(V600E) mutant, BRAF(V600E/K483M) was used as control.
(B) The non-canonical APE motif decreases the dimer affinity of BRAF(V600E) but can not completely abolish its dimerization. FLAG- and HA-tagged BRAF mutants were co-expressed in 293T cells that were lyzed with RIPA buffer containing 0.1% NP-40, and the co-immunoprecipitation assay was carried out with a gentle wash of PBS as in Figure S5.
All images are representative of at least three independent experiments.

Supplementary Materials and Methods

To knockdown CRAF in braf$^{-/-}$ MEFs that stably express ARAF(ΔQA), shRNAs were designed by using a website software (http://katahdin.cshl.org/siRNA/RNAi.cgi?type=shRNA), and the lentiviral vectors were constructed by using traditional molecular cloning methods. The targeting sequences were listed as follow:

shRNA#1 5- GGAATGGAATGAGCTTACA-3

shRNA#2 5- GGAATGAGCTTACATGACT-3

Reference

1. Chakraborty R, Burke TM, Hampton OA, Zinn DJ, Lim KP, Abhyankar H *et al*. Alternative genetic mechanisms of BRAF activation in Langerhans cell histiocytosis. Blood 2016; 128: 2533-2537.

2. Chen SH, Zhang Y, Van Horn RD, Yin T, Buchanan S, Yadav V *et al*. Oncogenic BRAF Deletions That Function as Homodimers and Are Sensitive to Inhibition by RAF Dimer Inhibitor LY3009120. Cancer Discov 2016; 6: 300-315.

3. Estep AL, Palmer C, McCormick F, Rauen KA. Mutation analysis of BRAF, MEK1 and MEK2 in 15 ovarian cancer cell lines: implications for therapy. PLoS One 2007; 2: e1279.

4. Foster SA, Whalen DM, Ozen A, Wongchenko MJ, Yin J, Yen I *et al*. Activation Mechanism of Oncogenic Deletion Mutations in BRAF, EGFR, and HER2. Cancer Cell 2016; 29: 477-493.

5. Hu J, Stites EC, Yu H, Germino EA, Meharena HS, Stork PJ *et al*. Allosteric activation of functionally asymmetric RAF kinase dimers. Cell 2013; 154: 1036-1046.

6. Hu J, Ahuja LG, Meharena HS, Kannan N, Kornev AP, Taylor SS *et al*. Kinase regulation by hydrophobic spine assembly in cancer. Mol Cell Biol 2015; 35: 264-276.
